# Editorial: MALDI-TOF MS Application for Susceptibility Testing of Microorganisms

**DOI:** 10.3389/fmicb.2020.568891

**Published:** 2020-11-04

**Authors:** Karsten Becker, Sören Schubert

**Affiliations:** ^1^Friedrich Loeffler-Institute of Medical Microbiology, University Medicine Greifswald, Greifswald, Germany; ^2^Faculty of Medicine, Max von Pettenkofer Institute of Hygiene and Medical Microbiology, Ludwig Maximilians University Munich, Munich, Germany

**Keywords:** mass spectometry, MALDI-TOF, susceptibility testing, carbapenamase, resistance detection, MRSA - Methicillin-resistant *Staphylococcus aureus*

In the present era of multi-/pan-resistant microorganisms, the acceleration of the microbiological diagnostics is the most pressing task and declared aim of many academic and industrial research groups. These efforts are of fundamental importance for appropriate antibiotic treatment and efficient infection control measures. Besides neglected issues of the preanalytical processes, rapid identification, and rapid susceptibility testing are essential components to be addressed (van Belkum et al., 2013, 2019; Idelevich et al., 2018a). Conventional standardized methods for susceptibility testing are usually accurate, but require long incubation times, especially for fungi (Idelevich and Becker, 2015). In the past decades the primary emphasis was on the development of nucleic acid-based molecular assays, which are only partially able to meet these demands. In particular, all DNA-based molecular assays are focused to the respective resistance genes. The presence of these, however, does not necessarily correspond to the resistance phenotype, nor is applicable in case of unknown resistance mechanisms. Consequently, universal approaches are necessary, which allow for (i) rapid and untargeted identification, (ii) fast and mechanism-independent susceptibility testing and, if needed, (iii) further characterization (e.g., typing) of a given isolate.

Although being a phenotypic approach, matrix-assisted laser desorption/ionization time-of-flight mass spectrometry (MALDI-TOF MS)-based analysis of the whole-organism protein mass spectra offers the closest approximation to the ribosomal sequence information. This method has been successfully established for the identification and subtyping of bacterial and fungal microorganisms (Wieser et al., 2012; Clark et al., 2013) and, meanwhile, it has become a routine method in many diagnostic laboratories contributing enormously to a reduction of the time-to-result in identification procedures (Idelevich et al., 2019). However, for susceptibility testing, rapid phenotypic approaches feasible for routine applications are still missing (Schubert and Kostrzewa, 2017). As shown in this special issue summarizing the results of the respective Frontiers Research Topic “MALDI-TOF MS Application for Susceptibility Testing of Microorganisms,” this technology is able to provide solutions for rapid determination of antibiotic susceptibilities independently of the underlying resistance phenotype.

The review article by Burckhardt and Zimmermann provides an excellent entrance into the different MALDI-TOF MS approaches for specific or universal determination of antimicrobial resistances and addresses the question why we do need timely susceptibility testing. This overview is complemented by a mini-review by Florio et al. about the detection of antifungal resistance by MALDI-TOF MS. Approaches to determine bacterial and fungal resistances comprise technical solutions using (i) defined marker peaks to deduce susceptibility or resistance, (ii) alterations of antibiotics such as hydrolysis, decarboxylation or acetylation as read-out, (iii) peak shifts as read-out by incorporation of ^13^C, (iv) quantification of the area under the curve as read-out and, as newest development, (v) the direct-on-target microdroplet growth assay (DOT-MGA).

The latter approach, originally described by Idelevich et al. (2018b), is represented in this issue by two articles reporting on the detection of methicillin resistance in *Staphylococcus aureus* by Nix et al. and on the rapid detection of extended-spectrum β-lactamases and AmpC β-lactamases in *Enterobacterales* by Correa-Martínez et al. Instead of targeting specific resistance mechanisms, it resembles the minimum inhibitory concentration (MIC) determination by the broth microdilution method by using MALDI-TOF MS targets directly as incubation device. Thus, the created layout of the test panel as well as the interpretation criteria applied follow the criteria of the European Committee on Antimicrobial Susceptibility Testing (EUCAST). Both articles demonstrated that these novel phenotypic methods are able to provide reliable results in a short time.

Dortet et al. present results on another innovative derivative of the MALDI-TOF MS technology, the so-called MALDIxin test, which has been recently developed by this group (Dortet et al., 2018). By directly assessing lipid A modifications in intact bacteria, this approach is able to identify polymyxin-resistant isolates in about 15 min and owns the capacity to discriminate between chromosome- and plasmid-encoded resistance as further advantage. Here, the authors showed their results of applying the MALDIxin to detect colistin-resistant *Salmonella enterica* isolates.

Further articles report on the evaluation of already existing commercially available MALDI-TOF mass spectrometry kits. Recent EUCAST guidelines for carbapenemase detection (European Committee for Antimicrobial Susceptibility Testing of the European Society of Clinical Microbiology Infectious Diseases, 2017) recommends hydrolysis assays by MALDI-TOF MS as a useful method for clinical practice. Several working groups evaluated the performances of the hydrolysis-based MBT STAR®-Carba IVD assay to detect carbapenemase-producing bacteria. The study of Cordovana et al. investigated a huge collection of diverse Italian and German *Klebsiella pneumoniae* isolates for the presence of the *Klebsiella pneumoniae* carbapenemases (KPC)-related peak, which has been confirmed in a subset by applying the MBT STAR®-Carba IVD assay. The confirmation procedure showed 100% sensitivity and specificity, both from colonies and from positive blood cultures. Using colony material, the assay as performed by Anantharajah et al. detected all included carbapenemase-producing *Enterobacteriaceae, Pseudomonas*, and *Acinetobacter* isolates with sensitivities and specificities of 100%. Of note, performed on positive blood cultures, the assay missed a few carbapenemase-producing *Acinetobacter* isolates, but a prolonged imipenem-incubation time of the strain pellet was able to improve the carbapenemase detection. Analyzing retrospective cultured isolates and prospective patient-derived blood cultures, Lee et al. measured the β-lactamase activities against various β-lactams including meropenem using the MBT STAR-BL module. Of interest, the assay protocol used enabled the reporting of β-lactamase-producing Gram-negative rods at ~14 and 48 h before the interim and final reports of routine BCs processing, respectively. Oviaño et al. could show that this assay was also able to reliably detect the activity of imipenem/relebactam, a novel β-lactam-β-lactamase inhibitor combination, with a turnaround time of less than 1 h in clinical *Enterobacterales* KPC isolates.

Another facets of MALDI-TOF MS are presented by Hu et al. and Wang et al.. Hu et al. intended to evaluate an automatic *S. aureus* subtyping module, which identifies highly specific the methicillin resistance of phenol soluble modulin (PSM)-bearing MRSA through detection of a specific PSM-*mec* peak by MALDI-TOF MS. Wang et al. presented a machine learning approach to generate robust heterogeneous vancomycin-intermediate *Staphylococcus aureus* (hVISA) detection models for analyzing the complex MALDI-TOF mass spectra.

In summary, this special issue gives the reader a comprehensive overview of the state of the art of MALDI-TOF MS applications on antimicrobial susceptibility testing as well as very recent insights in cutting-edge developments. Future tasks comprise the extension of MALDI-TOF MS-targeted antimicrobial substances, the continued transfer of research results into commercial applications including their thorough evaluation and improved strategies to integrate these applications in routine diagnostics and automation solutions.

## Author Contributions

KB and SS managed the Research Topic MALDI-TOF MS Application for Susceptibility Testing of Microorganisms as topic editors. KB wrote the manuscript with input from SS. All authors reviewed and edited the manuscript.

## Conflict of Interest

KB is inventor of a patent application which is owned by the University of Münster and licensed to Bruker Daltonik GmbH. The remaining author declares that the research was conducted in the absence of any commercial or financial relationships that could be construed as a potential conflict of interest.
